# Impact of FSH‐Induced Ovarian Stimulation on Oocyte Recovery and In Vitro Embryo Production in the Red Sindhi Cows

**DOI:** 10.1111/rda.70170

**Published:** 2026-01-08

**Authors:** Ivo Pivato, George Henrique Lima Martins, Lucas Costa de Faria, Heidi Christina Bessler, Carlos Frederico Martins

**Affiliations:** ^1^ Department of Animal Science University of Brasília Brasília Distrito Federal Brazil; ^2^ Department of Animal Health University of Brasília Brasília Distrito Federal Brazil; ^3^ Technology Center for Dairy Zebu Breeds from Embrapa Cerrados Planaltina Distrito Federal Brazil

**Keywords:** assisted reproduction, coasting, in vitro fertilisation, zebu

## Abstract

Follicle‐stimulating hormone (FSH) plays an important role in regulating reproductive events, particularly follicular development and oocyte competence acquisition. Some studies using FSH protocols in zebu cattle were performed, but data regarding its application in the Red Sindhi breed are scarce and therefore warranted. In this context, this study aimed to assess the FSH administration regimen—multiple doses (T1‐m) and single dose (T2‐s) and coasting period (54 h vs. 102 h) in oocyte developmental competence in Sindhi females. A total of 80 mg of FSH was administered either as a single application (40 mg IM + 40 mg, SC) or compared to multiple applications (30 mg + 30 mg + 20 mg, IM). Animals that did not receive FSH treatment serve as controls (CT). The present data showed that both T1 and T2 applications resulted in a greater number of medium‐sized follicles (7.80 vs. 8.57, *p* < 0.05), oocyte recovery (9.76 vs. 9.81), when compared to control (5.20; 6.30, respectively). Animals from T2 also had a greater number of aspirated follicles (12.52 vs. 8.70, *p* < 0.05), viable oocytes (7.33 vs. 4.45, *p* < 0.05) and blastocyst rates (43.22% vs. 29.11%, *p* < 0.05) than control animals. Our results showed that a reduced dose of FSH both single and multiple applications enhance oocyte developmental competence. Moreover, a single application of FSH combined with a longer coasting period offers practical advantages, making this approach more attractive for Sindhi breeding programmes.

## 1 Introduction

The Red Sindhi is a zebu cattle breed ( *Bos indicus* ) originating from Pakistan, characterised by its red coat colour, medium body size, heat tolerance, rusticity and dual‐purpose potential for both meat and milk production (de Barros et al. 2020; Mello et al. 2016). In Brazil, favourable climatic conditions and the availability of high‐quality pasture and water have enabled the full expression of the breed's productive potential, making Red Sindhi cattle economically advantageous under national production systems (Panetto et al. 2017). Additionally, the breed is recognised for its high fertility and sexual precocity, which are important for promoting genetic enhancement and reducing generation interval (Mello et al. 2020). This emphasises the importance of studying and enhancing the efficiency of reproductive techniques in this breed.

In vitro embryo production (IVEP) has remained a central focus of reproductive biotechnology for many years, owing not only to its capacity to enhance reproductive efficiency in both males and females but also to its role in accelerating the dissemination of superior animals (Ferre et al. 2020). Numerous strategies have been investigated to improve oocyte developmental competence. A well‐established principle in the literature is the positive association between follicular size and the ability to yield a developmentally competent oocyte capable of successful ovulation and subsequent embryonic development (Lonergan et al. 1994). Consequently, several studies have evaluated ovarian superstimulation (OvS) achieved through multiple administrations of FSH prior to ovum pick‐up (OPU) as an approach to enhance blastocyst production and, ultimately, increase pregnancy rates (Vieira et al. 2014).

Follicle‐stimulating hormone (FSH) plays a central role in reproductive physiology, contributing to spermatogenesis in bulls and to follicular growth and the acquisition of oocyte developmental competence in cows (Roelen 2019). Considering this, numerous studies have evaluated ovarian stimulation protocols in conjunction with IVEP as a strategy to enhance the reproductive efficiency of oocyte donors (Blondin et al. 2002; Egashira et al. 2019; Hayden et al. 2023; Nivet et al. 2012; Ongaratto et al. 2020; Pinheiro et al. 2023). Lonergan et al. (1994) have suggested that the beneficial effects of FSH on embryo production are largely attributable to its ability to increase follicular diameter, which in turn is associated with improved oocyte recovery rates. Conversely, several studies have demonstrated a direct positive impact of FSH on oocyte quality itself, ultimately leading to higher blastocyst yields (Egashira et al. 2019; Hayden et al. 2023; Nivet et al. 2012; Ongaratto et al. 2020).

It has been suggested that mere stimulation through subsequent applications of FSH may not be sufficient to enhance oocyte competence. Blondin et al. (2002) proposed that a period of FSH deprivation, known as ‘coasting’, is essential to induce the necessary cellular changes in the oocyte to improve its potential to develop into an embryo.

In this context, many studies have aimed to determine the optimal interval between the last FSH application and OPU. For example, Nivet et al. (2012) tested four different coasting periods (20, 44, 68 and 92 h) and concluded that the most effective intervals were between 44 and 68 h. An interesting study in ewes demonstrated that a 60‐h coasting period improved oocyte quality and the expression of quality‐related genes (Pinheiro et al. 2023).

Research investigating optimal hormonal stimulation protocols and coasting durations in Red Sindhi cattle remains limited. Therefore, the present study aimed to assess the effects of single versus multiple FSH administrations and two coasting intervals (54 or 102 h) on oocyte quality and IVEP in this breed.

## 2 Materials and Methods

All experimental procedures were performed in accordance with current Brazilian laws and were approved beforehand by the ethics committee on animal use at the University of Brasilia (UnB/DOC no 13522/2013).

### 2.1 Location

This study was performed at the Center of Technology for Zebu Dairy Breeds (CTZL) from Embrapa Cerrados, located in Brasilia, Federal District, Brazil (15°57′09″ S and 48°08′12″ W). This region represents part of the Cerrado (savannah) biome found in central Brazil and is the second largest national biome with 204 million ha, representing 24% of the country's area. The annual average rainfall ranges from 800 to 1800 mm. Climatic seasons are well defined, with dry winters and rainy summers. The climate is tropical, with average temperatures of 22°C–23°C (Sano et al. 2019).

### 2.2 Experimental Design

In the present study, 12 non‐lactating and non‐pregnant Sindhi cows were used. All animals presented a good body score condition (3.70; 1–5 scale), a mean weight of 400 kg and 6 years old. The animals were kept in a grazing system, in a rotated pasture composed of *Panicum maximum Jaca. vr Mombaça*, with water and mineral supplementation ad libitum during the experiment.

**FIGURE 1:**
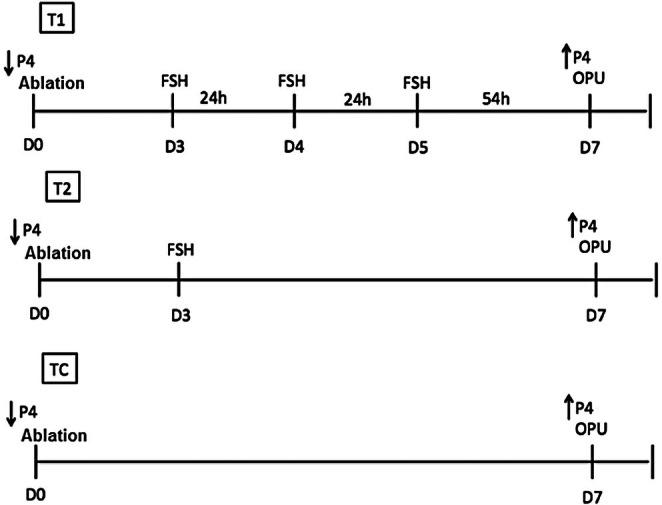
Schematic representation of experimental design.

At the beginning of the experimental period, all animals were examined for the presence of a dominant follicle (DF). Follicles larger than 5 mm were aspirated to achieve dominant follicle removal (DFR). Subsequently, immediately after DFR, all females were synchronised in day 0 (D0) using an intravaginal progesterone device (1 g; Cronipres, Biogénesis‐Bagó S.A., Buenos Aires, Argentina), which remained in place until OPU in day 7 (D7). Three experimental treatments were evaluated: Treatment 1 (T1‐m): DFR and application of FSH in multiple doses (*n* = 4 females). In this treatment, OvS consisted of 80 mg FSH (Folltropin‐V; Bioniche, Canada, containing pituitary extract of porcine follicle‐stimulating hormone (pFSH) 400 mg NIH) administered intramuscularly in three decreasing doses (30 mg, 30 mg and 20 mg) at 24‐h intervals between each application. The first FSH application was commencing from day 3 (D3) of progesterone device application, and the last application was administered on day 5 (D5), 54 h prior to OPU; Treatment 2 (T2‐s): DFR and application of FSH in a single dose (*n* = 4 females). In this treatment, OvS consisted of the same total FSH dose (80 mg), applied as a single dose commencing from day 3 (D3) of progesterone device application, with 40 mg administered intramuscularly and 40 mg subcutaneously, 102 h prior to OPU; Control treatment (CT): DFR and OPU (*n* = 4 females): animals underwent DFR and the OPU without ovarian stimulation. After OPU, all animals received a new intravaginal implant and a new replicate was started. Five replicates were performed for each treatment within an interval of 7 days between each OPU‐hormonal stimulation, as shown in Figure 1.

The evaluations to determine the effects of each treatment were divided into two stages: Stage 1—Follicular Assessment: this phase evaluated the effects of single versus multiple FSH applications on follicular population and size distribution. All visible follicles were counted and measured using a 7.5 MHz linear‐array transducer (Honda, Japan) connected to an HS 1500 ultrasound unit (Honda, Japan). Follicles were classified based on diameter as follows: small (< 3 mm), medium (3–8 mm) and large (> 8 mm); Stage 2—IVEP Evaluation: in this phase, the influence of FSH treatment protocol on IVEP was evaluated. Females from all treatments were subjected to OPU and recovered cumulus–oocyte complexes (COCs) were evaluated and subsequently processed for in vitro maturation (IVM), fertilisation (IVF) and embryo culture (IVC). In this phase, the following variables were recorded: number of follicles aspirated, total oocytes recovered, number of viable oocytes, cleavage rate and blastocyst yield on day 7 of culture.

### 2.3 Ovum Pick‐Up

The OPU procedure was conducted using an HS 1500 ultrasound unit (Honda, Japan) equipped with a sectorial, multi‐frequency (5–10 MHz) micro‐convex probe (HCV‐3710 MV, Honda, Japan) mounted on a transvaginal aspiration guide (WTA, Brazil). Follicular aspiration was performed using an 18G needle (WTA, Brazil) connected to a digital vacuum pump (BV‐003d, WTA, Brazil), with the aspiration flow rate maintained at 15 mL/min.

### 2.4 In Vitro Embryo Production (IVEP)

IVEP procedures followed the methodology described by de Toledo et al. (2023), with minor modifications. Recovered oocytes were morphologically classified into four grades (I–IV) according to the number of layers of cumulus–oophorus cells, cumulus–oophorus expansion, cytoplasmic homogeneity, colour and structural integrity. Only Grade I, II and III COCs were selected for IVM.

For IVM, COCs from each donor were placed in 200 μL of maturation medium (MIV), consisting of TCM‐199 with Earle's salts (Thermo Fisher Scientific Inc.) supplemented with 10% fetal bovine serum (FBS), LH (24 IU/mL), FSH (10 μg/mL) and antibiotics. Maturation was carried out for 22 h at 38.5°C in a 5% CO_2_ atmosphere. Matured COCs were allocated in groups of 30 oocytes into 150 μL drops of fertilisation medium (TALP; Tyrode's albumin lactate pyruvate) supplemented with 2 mM penicillamine, 1 mM hypotaurine, 250 μM epinephrine and 10 μg/mL heparin (Sigma). Frozen–thawed semen was evaluated for viability and subjected to selection via a 45%–90% Percoll density gradient (Sigma Aldrich). The final sperm concentration in fertilisation drops was adjusted to 1 × 10^6^ spermatozoa/mL. Fertilisation was performed for 18 h at 38.5°C under 5% CO_2_.

For in vitro culture (IVC), presumptive zygotes were partially denuded and transferred to synthetic oviduct fluid (SOF) supplemented with 0.34 mM sodium tricitrate, 2.77 mM myo‐inositol (Sigma Aldrich) and 5% FBS. Embryo development was evaluated at 44 h post‐insemination to determine cleavage rate and on day 7 (D7) to assess blastocyst production.

### 2.5 Statistical Analysis

The study was entirely casualised with three treatments and five repetitions, considering in statistical model the fixed effect of treatments on the variables: total number of follicles aspirated, the number of follicles in each size category, total number of COCs recovered and viable COCs per cow, as well as cleavage rate (number of cleaved zygotes per total number of COCs cultured), blastocyst rate (number of blastocysts produced per total number of COCs cultured) and number of embryos produced per OPU procedure.

Firstly, data normality was verified by the Shapiro–Wilk test. The variation in the follicle size before OPU, number of follicles, recovered and viable COCs among OPU sessions was determined by ANOVA variance test. When ANOVA detected statistical differences, the Tukey test was used to compare all treatments. Binomial variables such as cleavage and blastocyst rates were compared using the Chi‐squared method. Results are shown as mean ± SEM and statistical significance was determined based on a *p* value < 0.05.

## 3 Results

### 3.1 Stage 1—Follicular Growth

There were substantially more medium follicles (*p* < 0.05) in both OvS treatments than in the control treatment. No differences were observed concerning small and large follicles among treatments (Table 1).

**TABLE 1:** Number of follicles by diameter category (mean ± SEM) per Sindhi cow stimulated with multiple (T1‐m) or single (T2‐s) FSH application.

Treatment	Follicle size		
	< 3 mm	3–8 mm	> 8 mm
T1 (m)	0.52 ± 0.17	7.80 ± 0.80ª	1.20 ± 0.23
T2 (s)	0.76 ± 0.24	8.57 ± 1.04ª	1.38 ± 0.24
Control	0.80 ± 0.33	5.20 ± 0.51^b^	1.50 ± 0.19

*Note:* Different letters “^ab^” in the same column indicate differences between groups (*p* < 0.05). Control: without FSH stimulation (control).

### 3.2 Stage 2—In Vitro Embryo Production

There was no observed difference in the mean number of COCs recovered between T1 (m) and T2 (s); however, OvS treatments presented more COCs than the control treatment (*p* < 0.05; Table 2). The number of viable oocytes was similar between treatments with hormonal stimulation, but only the single hormonal application treatment presented more viable oocytes than the control treatment (*p* < 0.05; Table 2).

**TABLE 2:** Number of aspirated follicles, recovered oocytes and viable oocytes per Sindhi cow (mean ± SEM) stimulated with multiple (T1‐m) or single (T2‐s) FSH application.

Treatment	Aspirated follicles (*n*)	Retrieved oocytes (*n*)	Viable oocytes (*n*)
T1 (m)	11.36 ± 1.39^ab^	9.76 ± 1.32ª	6.72 ± 1.04^ab^
T2 (s)	12.52 ± 1.81ª	9.81 ± 1.83ª	7.33 ± 1.32ª
Control	8.70 ± 0.74^b^	6.30 ± 0.82^b^	4.45 ± 0.51^b^

*Note:* Different letters “^ab^” in the same column indicate differences between groups (*p* < 0.05). Control: without FSH stimulation (control).

Single dose of FSH treatment presented a lower rate of cleavage than multiple doses of FSH treatment (84.75% vs. 93.52%, respectively, Table 3).

**TABLE 3:** Cleavage and blastocysts rates (mean ± SEM) in Sindhi cows stimulated with multiple (T1‐m) or single (T2‐s) FSH application.

Treatment	Oocytes (*n*°)	Cleavage (%)	Blastocyst D7 (%)
T1‐m	108	93.52 ± 4.19 (101/108)^a^	38.89 ± 0.65 (42/108)^a,b^
T2‐s	118	84.75 ± 5.79 (100/118)^b^	43.22 ± 0.48 (51/118)^a^
Control	79	86.08 ± 2.94 (68/79)^a,b^	29.11 ± 0.95 (23/79)^b^

*Note:* Different letters “^ab^” in the same column indicate differences between groups (*p* < 0.05). Control: without FSH stimulation (control).

**FIGURE 2:**
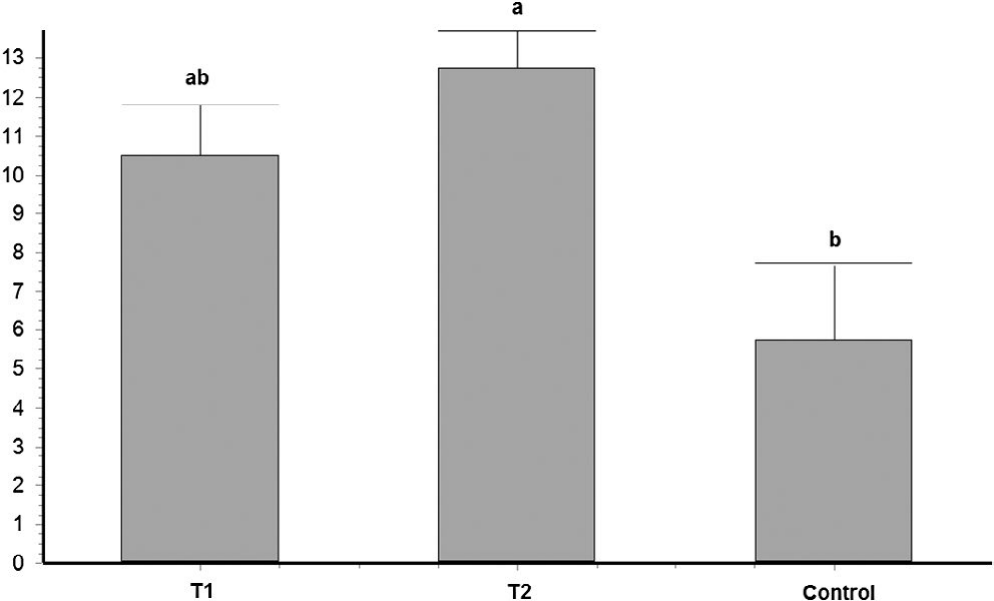
Embryo production/OPU (mean ± SEM) of animals in the different treatments.

Regarding embryo production, it was observed that follicle stimulation using a single dose of FSH (T2‐s) reflected in a better blastocyst rate in D7 when compared to the non‐treated group (43.22% vs. 29.11% of embryos in T2‐s and TC, respectively; *p* < 0.05). These results reflect a difference of 45% in embryo production per OPU between the treatment that used a single application of FSH and 102 h of coasting compared to the control treatment (12.75 ± 0.95 vs. 5.75 ± 1.9 embryos/OPU, respectively for T2‐s and TC; Figure 2).

No differences were observed between T1‐m and T2‐s treatments (*p* > 0.05; Table 3).

## 4 Discussion

In our experiment, the use of a reduced concentration of FSH in single and multiple dosages induced an enhancement in the number of follicles and their size, oocyte viability and, consequently, embryo production rates, reinforcing its importance as a reproductive tool. A single application of FSH in the Red Sindhi females and 102 h of coasting produced about 45% more embryos per OPU than the control treatment.

It is well known that FSH plays an important role in reproductive biology in mammals, inducing follicular growth in females and participating actively in oocyte developmental acquisition (Roelen 2019). When its use becomes prolonged, such as in OvS protocols, it is expected that more follicles pass through these steps, resulting in more than one follicle reaching the dominant stage. Our results showed that the use of FSH in single and multiple applications enhanced the number of medium follicles (3–8 mm; *p* < 0.05), highlighting the importance of FSH supplementation on follicle development. Other studies using ovarian stimulation found a similar pattern of follicular growth. When Blondin et al. (2002) tested two different protocols of OvS with 4 or 6 FSH applications and distinct coasting intervals (33 and 48 h) before OPU, they observed that the Holstein heifers treated with FSH, independent of coasting interval, presented more follicles (> 3 mm) than the non‐treated group. Furthermore, Hayden et al. (2023) also observed that the use of FSH in multiple dosages resulted in a higher number of medium follicles (6–10 mm) in pregnant Holstein heifers than large or small follicles.

In females treated with FSH, the increase in follicle count was directly associated with a higher number of oocytes recovered, indicating a positive correlation between follicular development and oocyte yield (9.76 and 9.81 vs. 6.3 COCs/OPU/cow, respectively, for T1 (m), T2 (s) and CT). It has been described in the literature that puncture of follicles larger than 4 mm results in a minor COCs recovery rate (Seneda et al. 2001), conversely, the relation between follicle size and its recovery capability is controversial. Comparing the effect of a single application of FSH in Japanese cattle, Egashira et al. (2019) described that, even though there was an increase in the size of the follicles, no difference was observed in oocyte recovery, suggesting that the follicle size had no effect. The same was observed in pregnant Holstein heifers by Hayden et al. (2023). Our results contrast with these findings, and a possible explanation is based on the increase in the number of follicles in proportion to the increase in their size in the ovaries of stimulated Red Sindhi cows, which could result in a higher number of recovered structures.

The use of FSH in single and multiple applications had a positive impact in oocyte viability by enhancing oocyte morphological quality in our study. This finding contrasts with the results reported by Hayden et al. (2023), who observed that multiple FSH applications in pregnant heifers did not impact oocyte viability. Along with the differences in morphological quality, the presented results showed that the use of FSH promoted an improvement of oocyte competence by increasing the embryo production rate by approximately 15%. Despite the lower cleavage rate observed with the single‐dose FSH treatment compared to the multiple‐dose and control groups, this protocol led to a higher yield of blastocysts by day 7. Many authors reported that the use of superstimulation indeed enhances blastocyst development (Egashira et al. 2019; Hayden et al. 2023; Nivet et al. 2012; Vieira et al. 2014; Vieira et al. 2016), reinforcing its importance as a reproductive tool in embryo production efficiency.

Our results also demonstrated that a single application of FSH is efficient in inducing an increase in follicle number, oocyte viability and embryo production rates, as in the use of multiple applications of FSH, but could be a better strategy due to the reduced animal management. In contrast, although a single FSH administration influenced ovarian response, Chaubal et al. (2007) reported that multiple FSH applications improved ovarian efficiency in crossbred Angus cows prior to OPU and led to higher blastocyst rates compared to both single administration and control groups. *Bos indicus* cattle are known to be more sensitive to exogenous gonadotropin administration (Baruselli et al. 2006) than *Bos taurus* breeds. Studies of OvS in the Sindhi breed are still scarce. Some authors report that a lower dose (100 mg) in multiple applications had a successful effect in inducing multiple ovulations in primiparous and nulliparous Sindhi donors (Mattos et al. 2011). A study performed by Carvalho et al. (2010) assessed the use of different concentrations (100, 133 and 200 mg) of FSH in Sindhi superstimulatory response. Despite the better response indicated by the higher number of corpora lutea (CL) with the 200 mg dose, there was a lower recovery rate of structures through uterine flushing compared to the other dose groups. A possible explanation is that higher FSH doses may increase the number of oocytes that remain trapped in follicles, which luteinise instead of ovulating (Clark et al. 2022). In the present study, all Sindhi donors underwent weekly hormonal stimulation, which could increase the risk of endocrine and metabolic disorders. To minimise these risks, a lower FSH dose (80 mg) was used. Furthermore, we opted to use FSH from porcine pituitary diluted in saline solution, as its half‐life is short in the bloodstream. According to Demoustier et al. (1988), pFSH has a relatively short elimination half‐life of approximately 5 h in circulating plasma, while the total clearance is estimated to be around 10–12 h.

In treatment 2, the use of a single dose of pFSH with a first intramuscular application and a second subcutaneous application, aimed to slightly prolong the action of FSH on ovarian follicles, as well as reduce animal handling on the farm, avoiding the application of multiple doses over several days. Since the replicates were weekly, we also did not use any carrier vehicle that would prolong the release of FSH for a longer time, such as hyaluronan, a biocompatible natural glycosaminoglycan, that has been used as a diluent to support the sustained release of various drugs (Oh et al. 2010).

Despite the reduced dosage and number of applications, all donors showed a satisfactory ovarian response. In this context, our study is the first to demonstrate the effectiveness of lower FSH doses and fewer applications in stimulating a favourable response in Sindhi cows.

## 5 Conclusions

According to our results, both single and multiple FSH doses significantly enhance oocyte developmental competence, leading to increased blastocyst yields. Moreover, a single FSH application combined with a longer coasting period simplifies livestock management and reduces stress, making this approach more practical and easier to implement.

## Author Contributions

**Ivo Pivato:** conceptualization, methodology, supervision and final review of the manuscript. **George Henrique Lima Martins:** methodology, investigation and data curation. **Lucas Costa de Faria:** writing, review, editing and visualisation. **Heidi Christina Bessler:** methodology, investigation and data curation. **Carlos Frederico Martins:** conceptualization, methodology, supervision, writing – original draft, final review of the manuscript, project administration and funding acquisition.

## Funding

This work was supported by Fundação de Apoio à Pesquisa do Distrito Federal, 00193‐0000002278/2022‐89.

## Conflicts of Interest

The authors declare no conflicts of interest.

## Data Availability

The data that support the findings of this study are available from the corresponding author upon reasonable request.
